# Supplementary material for: Impaired systemic antibody response against gut microbiota pathobionts in critical illness and susceptibility to nosocomial infections
Source: Intensive Care Med Exp. 2026 Jan 27;14:9. doi: 10.1186/s40635-026-00860-1 (PMC12847606; doi:10.1186/s40635-026-00860-1)
Supplement: Supplementary file 1 — Additional file1 (PDF 897 kb) [file 40635_2026_860_MOESM1_ESM.pdf]

Supplementary Material For:

Impaired systemic antibody response against gut microbiota pathobionts in critical illness and susceptibility to nosocomial infections

Nicole A. Cho, Jared Schlechte, Ian-ling Yu, Ish Bains, Tanner Fahlman, Colin Mackenzie, Braedon McDonald

SUPPLEMENTARY TABLES 1-3

SUPPLEMENTARY FIGURES 1-3

Supplementary Table 1. Characteristics of study participants.

**Characteristics**	**ICU patients (n=46)**	**Healthy volunteers (n=28)**
Demographics		
Age, median (range)	61 (24-86)	45 (29-74)
Female sex, *n* (%)	18 (39.1)	16 (57.1)
Male sex, *n* (%)	28 (60.9)	12 (42.9)
Comorbidities		
Diabetes, *n* (%)	8 (17.4)	N/A
Cardiovascular disease, *n* (%)	12 (26.1)	N/A
Chronic lung disease, *n* (%)	10 (21.7)	N/A
Cirrhosis, *n* (%)	0 (0)	N/A
Chronic kidney disease (on dialysis), *n* (%)	0 (0)	N/A
GERD, *n* (%)	9 (19.6)	N/A
Charlson index, median (range)	1 (0-8)	N/A
Admission diagnosis, *n* (%)		
Sepsis	21 (45.7)	N/A
Trauma	12 (26.1)	N/A
Neurological	9 (19.6)	N/A
Medical (other)	4 (8.7)	N/A
Illness severity		
Admission SOFA score, median (range)	8 (2-16)	N/A
Therapies, *n* (%)		
Invasive mechanical ventilation	46 (100)	N/A
Antibiotics at ICU admission	18 (39.1)	N/A
Enteral nutrition	46 (100)	N/A
Parenteral nutrition	0 (0)	N/A
Outcomes		
Nosocomial infection to day 30, *n* (%)	26 (56.5)	N/A
Duration of ventilation in days, median (range)	6 (1-21)	N/A
Duration of ICU stay in days, median (range)	7 (2-31)	N/A
Duration of hospitalization in days, median (range)	17 (4-207)	N/A
Mortality to day 30, *n* (%)	15 (32.6)	N/A

Supplementary Table 2. List of pathobiont microorganisms.

Organism	Strain	Source	Identifier
Staphylococcus aureus	ΔspaΔsbi SH1000	Gift from Bas Surewaard	N/A
Staphylococcus epidermidis	RP21	Alberta Microbiota Repository (AMBR)	N/A
Enterococcus faecalis	KB1	DSMZ	DSM 32036
Klebsiella aerogenes	Eaer1	Gift from Ian Lewis	N/A
Klebsiella oxytoca	K0x17	Gift from Ian Lewis	N/A
Klebsiella pneumoniae	ST258 (isolate no. KpCG02)	(Chan et al., 2013)	N/A
Enterobacter cloacae	Eclo25	Gift from Ian Lewis	N/A
Escherichia coli	ST131	(McNally et al., 2016)	N/A
Candida albicans	Clinical isolate	(Bernardes et al., 2024)	N/A
Pseudomonas aeruginosa	RP9	Alberta Microbiota Repository (AMBR)	N/A

Supplementary Table 3. Linear regression analyses of associations between patient characteristics, clinical measures, and antibiotics with antibody measurements.

**Variables**	**Models**					
	IgG anti-gut pathobiont MFI	IgM anti-gut pathobiont MFI	IgA anti-gut pathobiont MFI	Total IgG concentration in plasma	Total IgM concentration in plasma	Total IgA concentration in plasma
Age	-0.570 (0.26)	-0.688 (0.11)	0.961 (0.033)	0.180 (0.32)	-0.0227 (0.60)	0.365(0.10)
Admission SOFA	-0.0308 (0.91)	0.304 (0.21)	0.164 (0.52)	0.180 (0.81)	0.114 (0.52)	0.705 (0.43)
Sepsis	0.181 (0.20)	0.195 (0.10)	0.0995 (0.42)	-1.755 (0.77)	-0.777 (0.59)	-0.200 (0.98)
Charlson Index	0.0195 (0.67)	-0.0603 (0.12)	-0.072 (0.081)	-0.763 (0.70)	-0.336 (0.47)	-0.482 (0.84)
Antibiotic days prior to sample	-0.131 (0.18)	-0.0400 (0.62)	0.146 (0.087)	1.405 (0.73)	0.176 (0.86)	-0.0892 (0.99)

Data are Estimate (p value)

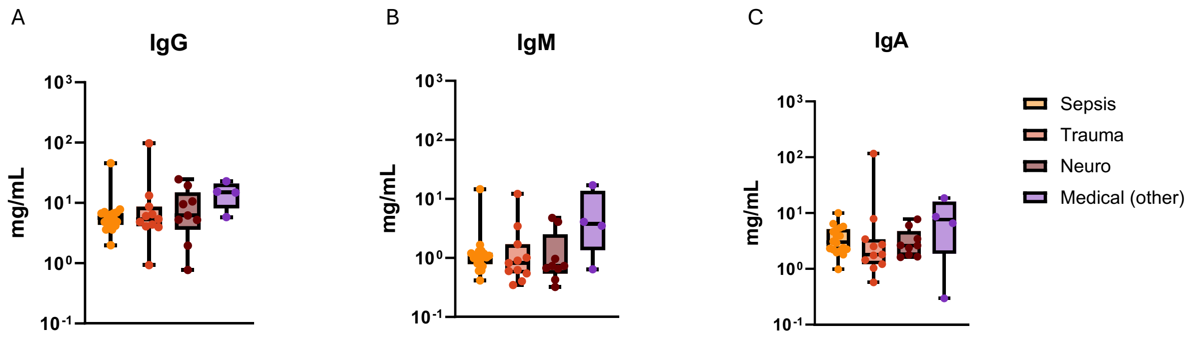

Supplementary Figure 1. Systemic antibody levels in ICU patients stratified by admission diagnosis. Quantitative analysis of (B) IgG, (C) IgM, and (D) IgA concentrations in the plasma of critically ill ICU patients (N=46) on day 1 of ICU admission, stratified by admission diagnosis. Dots represent individual patients, central line indicates median, box shows interquartile range (IQR) and whiskers show range; analyzed by Kruskal-Wallis test with a post hoc Tukey's test for multiple comparisons, p values non-significant ($p>0.05$).

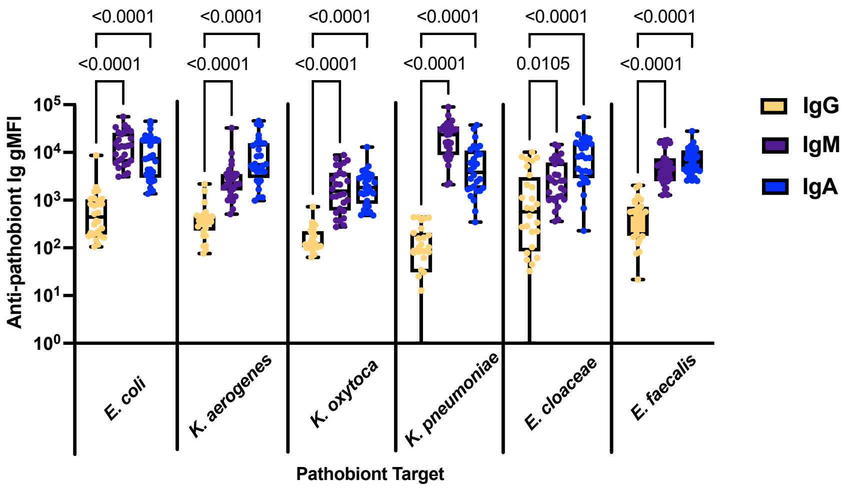

Supplementary Figure 2. Quantitative comparison of plasma IgG, IgM, and IgA binding to gut pathobionts in healthy volunteers. Flow cytometry was used to quantify plasma IgG binding to 6 individual gut pathobionts (expressed as median fluorescence intensity, MFI) in healthy volunteers (N=28). Dots represent individual patients, central line indicates median, box shows interquartile range (IQR) and whiskers show range; analyzed by pathobiont using Kruskal-Wallis test with a post hoc Tukey's test for multiple comparisons, significant p values as shown.

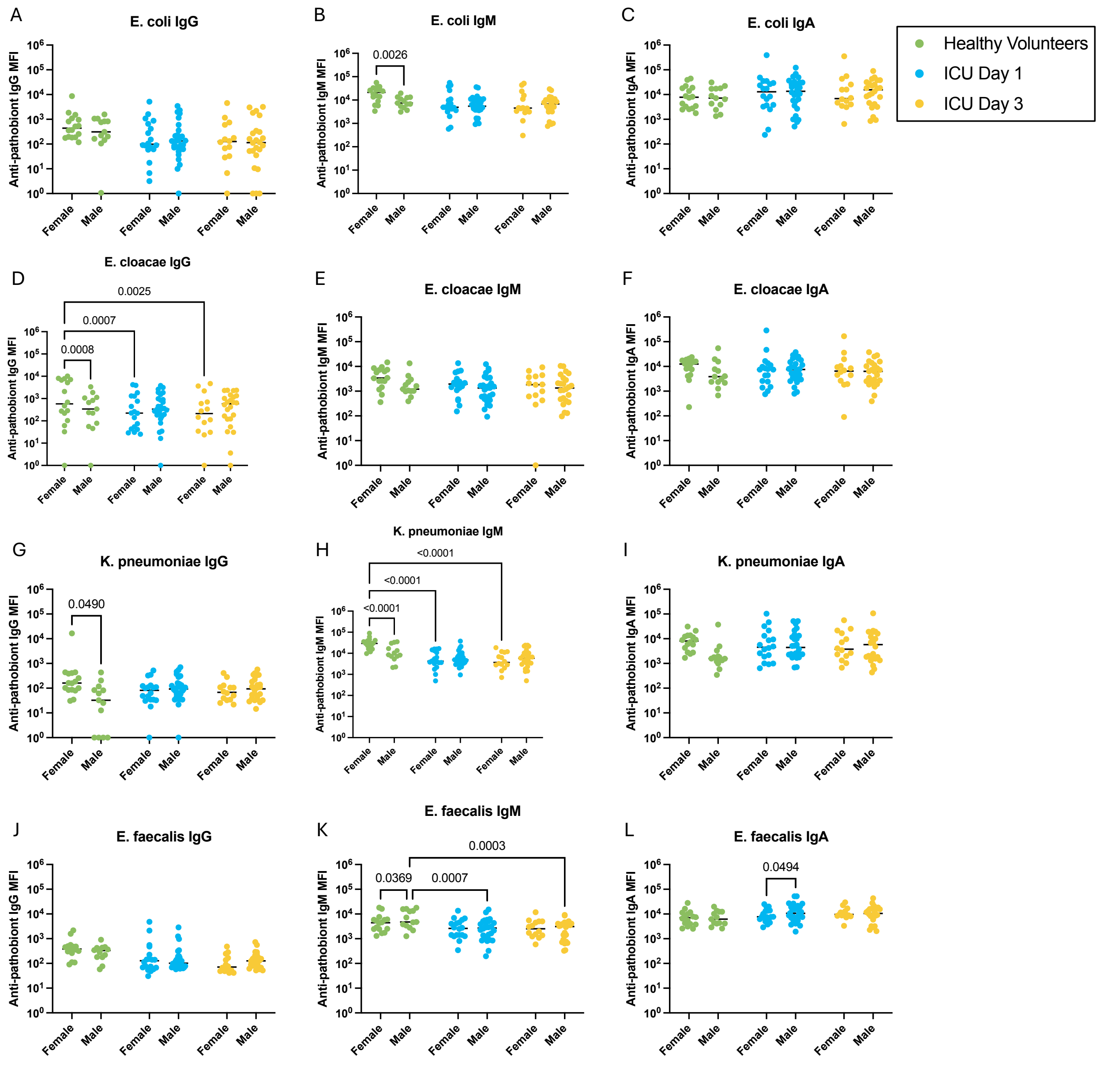

Continued on next page

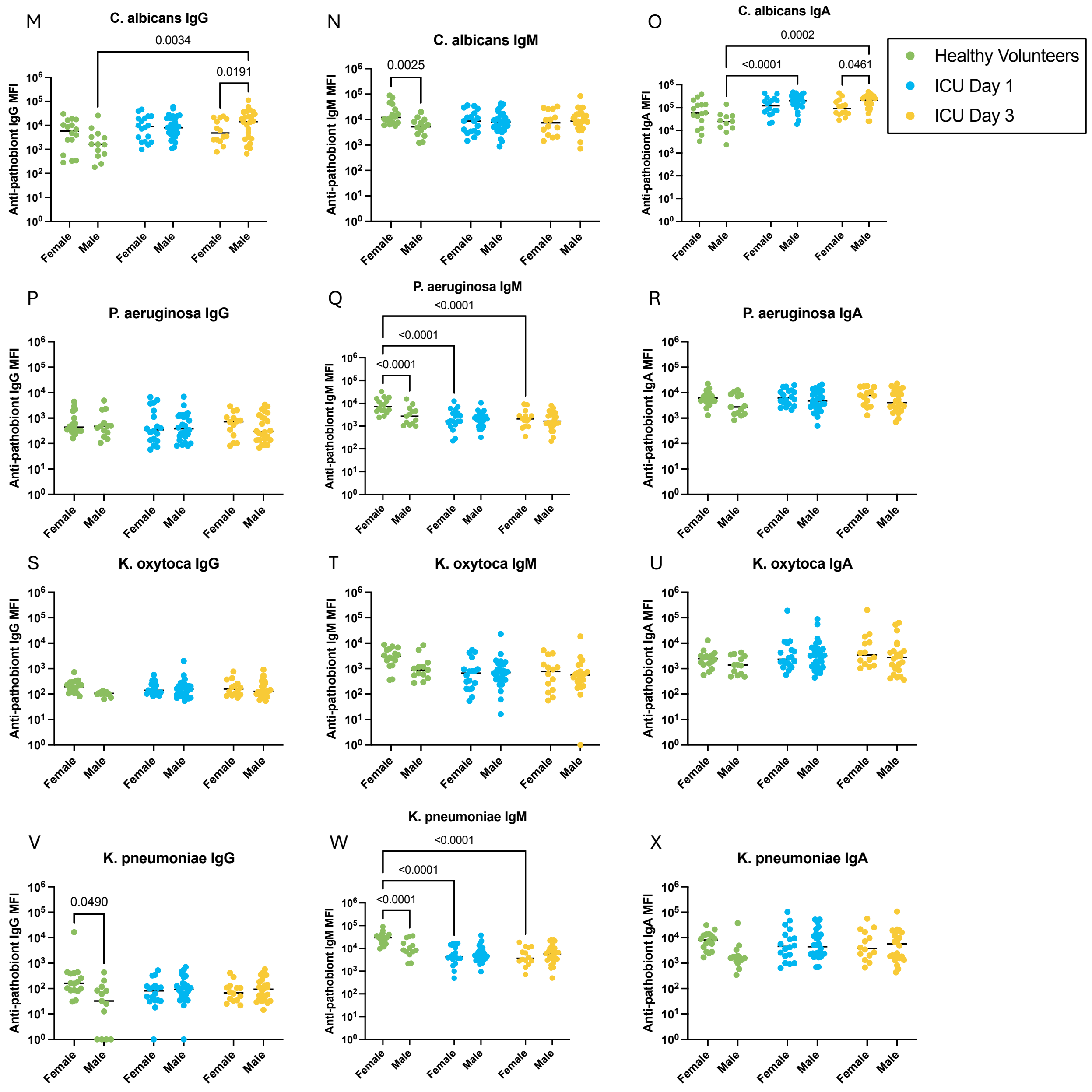

Continued on next page

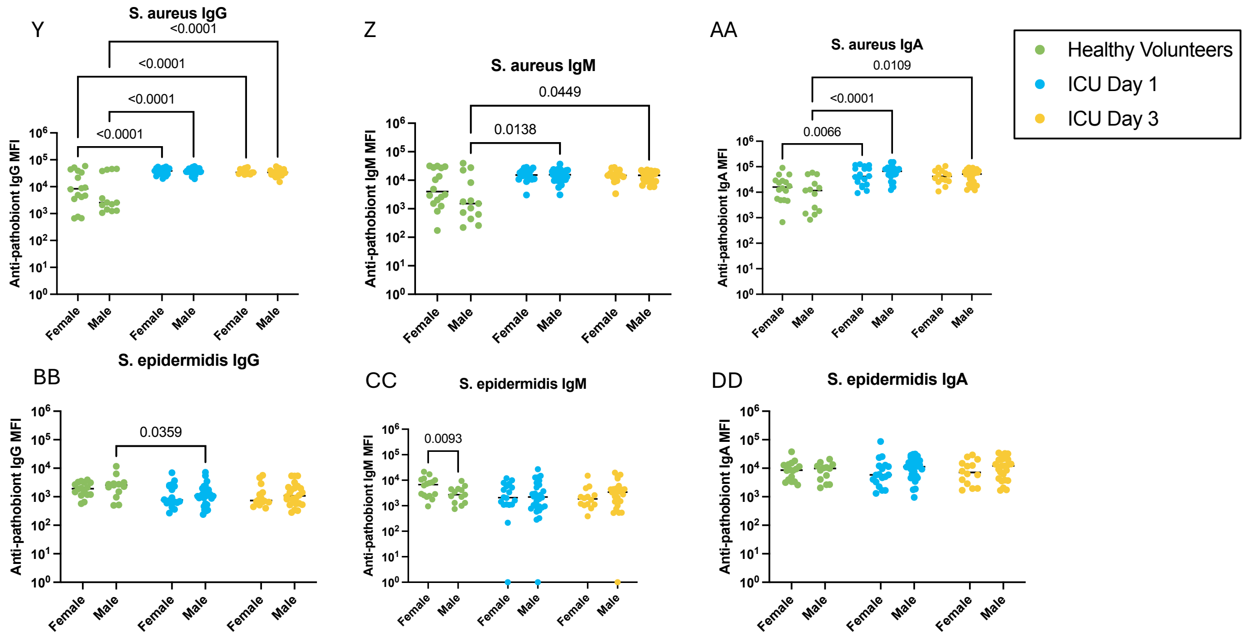

Supplementary Figure 3. Sex disaggregated analysis of systemic antibody responses against pathobionts in critically ill patients. Flow cytometry was used to quantify plasma IgG, IgM, and IgA binding to 10 pathobionts (isotype and pathobiont noted above each graph) expressed as median fluorescence intensity (MFI) in healthy (N=16 females, N=12 males) and critically ill patients (N=18 females, N=28 males) on days 1 and 3 of ICU admission. Dots represent individual patients, line indicates median; analyzed by Mixed-effects model with a post hoc Tukey's test for multiple comparisons, significant p values as shown.
